# Didgeridoo Health Promotion Method Improves Mood, Mental Stress, and Stability of Autonomic Nervous System

**DOI:** 10.3390/ijerph16183443

**Published:** 2019-09-17

**Authors:** Suni Lee, Shoko Yamamoto, Naoko Kumagai-Takei, Nagisa Sada, Kei Yoshitome, Yasumitsu Nishimura, Toshihiro Kojima, Takemi Otsuki

**Affiliations:** 1 Department of Hygiene, Kawasaki Medical School, 577 Matsushima, Kurashiki, Okayama 701-0192, Japan; slee@med.kawasaki-m.ac.jp (S.L.); s.yamamoto@med.kawasaki-m.ac.jp (S.Y.); kumagai@med.kawasaki-m.ac.jp (N.K.-T.); nagisada@okayama-u.ac.jp (N.S.); kei_y@med.kawasaki-m.ac.jp (K.Y.); yas@med.kawasaki-m.ac.jp (Y.N.); 2 Department of Biophysical Chemistry, Graduate School of Medicine, Dentistry and Pharmaceutical Sciences, Okayama University, Tsushima 1-1-1, Kita-Ku, Okayama 700-8530, Japan; 3 General Organization Corporation Didgeridoo Health Method Promotion Society, Hokan-cho 1-3-11, Kita-Ku, Okayama 700-8530, Japan; toshi@happydidgelife.com

**Keywords:** didgeridoo health promotion method, mood, POMS2, TMD (total mood disturbance), salivary amylase, autonomic nerve balance

## Abstract

A potential method of health promotion using the traditional wooden brass instrument the didgeridoo was examined, especially in terms of mood, stress, and autonomic nerve stabilization. Twenty Japanese healthy subjects undertook 10 lessons of the Didgeridoo Health Promotion Method (DHPM) and a moods questionnaire, blood pressure, salivary amylase (sAmy) as a stress marker, pulse rate and autonomic balance expressed by Ln[low frequency (LF)/High frequency (HF) were examined twice before the entire lessons and once before and after each lesson. The subjects had improved total mood disturbance (TMD: overall mood disorder degree) as measured by the Japanese version of the Profile of Mood States 2nd Edition (POMS2) as a result of taking the lessons. The pulse of the subjects decreased after the lessons, which correlated with a reduction in sAmy. Additionally, it was found that sAmy decreased after the lessons with increasing age of the subject, subjects with higher TMD before the lessons, or subjects with higher sAmy values before the lessons. With autonomic balance measured by Ln[LF/HF], subjects who had parasympathetic dominance as a result of the lesson were significantly more frequent. Additionally, it has been shown that Ln[LF/HF] decreased over 10 weeks, and it is also clear that the effect is sustained. Health promotion is an important concern for societies as a whole. In this study, it became clear that the DHPM affected mood, stress, and autonomic stability. Future studies should focus on monitoring the effects of continuing the lessons for a longer period of time. Additionally, physical effects such as strength of respiratory muscles should be examined. DHPM may be employed in the work place to promote the mental health of workers as well as in regional neighborhood associations/communities.

## 1. Introduction

Health promotion is one of the most important concepts with regard to the prevention of diseases caused by social and environmental circumstances [1,2,3]. Flynn described health promotion as a community problem-solving process [1]. However, its implementation is a major endeavor and begins with small group environments such as work places or regional neighborhood associations/communities to disseminate various health promotion methods. As reported by van Herten and van de Water, there are many targets and determinations regarding global health and health promotion [2]. Thus, it is important to think globally and to act locally, starting with small community groups to educate and train people in health promotion methods. Most people in developed countries are exposed to various stresses derived from social interactions in working environments and personal activities of ordinary lives [4,5,6].

However, there may be several issues such as costs for health promotion. McDaid and Park have referred to the marked costs involved in health promotion. It would be relatively easy in high-income countries, especially in the areas of mental health and well-being and/or primary prevention of poor mental health through health-related means. Joyce et al. have indicated that mental health and anxiety are some of the most important health problems in workplaces and that empirically supported interventions should be performed to maintain the mental well-being of workers [6]. Of course, health promotion depends on multiple factors.

Various governmental policies should be enacted and encouraged, such as efforts to increase the rate of medical checkups, legal measures to reduce mental stress at the workplace, and efforts to prevent diseases through health education [1,2,3]. Additionally, people need to possess an awareness and motivation for health promotion. It is important to improve the physical and mental well-being of citizens through daily activities and lifestyle habits that relieve stress. In particular, mood stabilization on a daily basis is very important, general methods need to be embraced that do not induce excessive mental stress, and efforts to stabilize autonomic nerves are required to help improve personal health.

Health promotion can be understood in terms of the enactment of primary prevention strategies. When focusing on mental health and emotional well-being, it is important that these strategies be performed continuously and without the requirement of considerable effort. Furthermore, more effective strategies might include contact with other individuals to facilitate the collective experience and enjoyment of, for example, music or art, or situations that relieve tension and stress. If a new strategy or method can be devised for health promotion, it is necessary to assess the efficacy of such a method by examining mood surveys, the degree of mental stress, and changes in the autonomic nervous system. On that basis, it is important to consider what types of daily life situations need to be introduced as part of the method to expand its usefulness to as many people as possible.

The didgeridoo is an Aboriginal brass instrument that is indigenous to the Australian continent. Although it is wooden, it is classified not as a woodwind instrument but as a brass instrument by the sound generation principle [7,8]. One of the authors, T. Kojima, has been mastering the traditional wooden brass instrument the didgeridoo since 2000, and has been active as a performer and performance leader. It has been acknowledged that the promotion of abdominal breathing, moderate stimulation of respiratory muscles, and stimulation such as overtones and vibration of sounds associated with didgeridoo performance may lead to health promotion. Additionally, in 2014, the Didgeridoo Health Method Promotion Society (DHMPS) was established, along with development of the didgeridoo health promotion method (DHPM), which includes a trainer of the DHPM referred to as a Meister, has been deployed nationwide in Japan.

Regarding the didgeridoo, several reports have been published in which the effects of didgeridoo playing on strengthening respiratory muscles were examined. Most of the subjects in these studies comprised patients with bronchial asthma or sleep apnea syndrome [9,10,11]. Puhan et al. assessed the effects of didgeridoo playing on daytime sleepiness and other outcomes related to sleep and found a reduction in collapsibility of the upper airways in patients with moderate obstructive sleep apnea syndrome and snoring [9]. They found that regular didgeridoo playing is an effective alternative treatment that is well accepted by patients with moderate obstructive sleep apnea syndrome [9]. Eley et al. focused on bronchial asthma since the prevalence of asthma in Australian Aboriginal people is relatively high. They found that playing the didgeridoo had a positive effect in controlling asthma [10,11].

In this study, we observed the effects of the DHPM (comprising playing practice as the “health promotion method” and including preparatory exercises) in terms of mood fluctuation, mental stress index, and autonomic nervous regulation. This study was performed since one of the authors (T. Kojima), a Meister of DHPM and didgeridoo player, heard many anecdotal accounts of students indicating that playing the didgeridoo leads to an increased feeling of relaxation and a general improvement of mood. Hence, we attempted to examine the effects of DHPM lessons on mood, stress and the autonomic nervous system. There has not been any research evidence found about the topic of this article.

**Figure 1:**
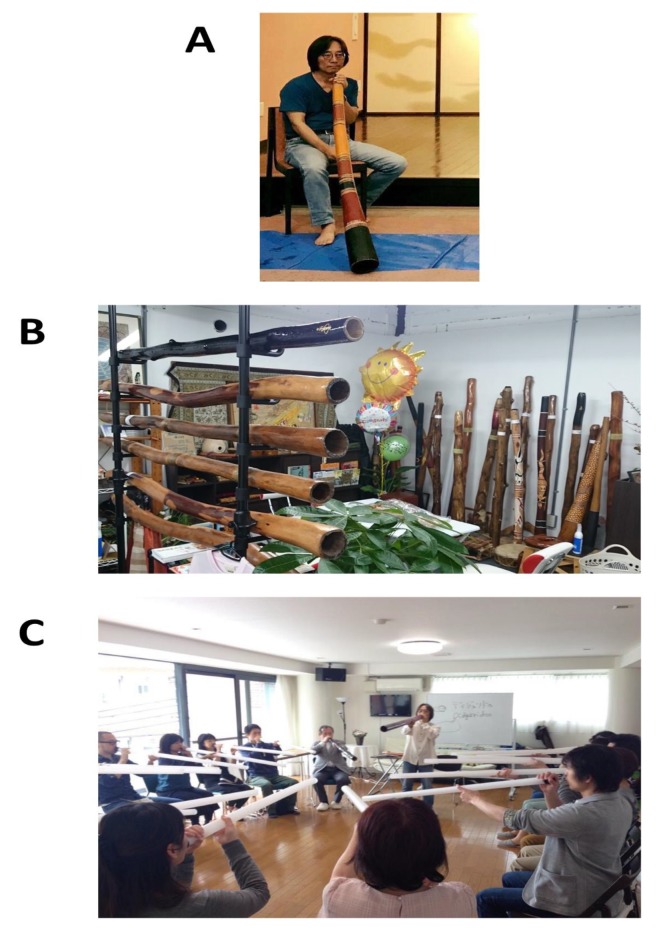
Didgeridoo performance and health promotion method classroom. (**A**) Just before playing the didgeridoo. (**B**) Various didgeridoos. (**C**) A lesson of the Didgeridoo Health Promotion Method in progress.

The DHPM classes were performed as described below. Figure 1A shows a didgeridoo being played by one of the authors, T. Kojima. Usually, didgeridoo is played while sitting down on the floor or on a chair. Several types of didgeridoo are introduced in Figure 1B. This instrument is the world’s oldest wooden instrument that Aboriginal natives, with a history of over 4000 years, used in ceremonies dating back to over 1000 years ago [7,8]. The didgeridoo is a brass instrument. Eucalyptus trees are cut off by termites and used as musical instruments, and each is colored as shown in the figure. It is positioned as any brass instrument to facilitate vibration of the lips and generate sound without using a reed. Additionally, as people become more proficient players, performance includes cyclical breathing—a breathing method that involves breathing in from the nasal cavity and is exhaled from the oral cavity and where there is no interruption in sound due to breathing, which can result in continuation of sound over long times.

As shown in Figure 1C, in the “DHPM” lesson held by the DHMPS Okayama branch and Meister training courses and at other locations by a Meister, plastic cylinders are used in lieu of the actual didgeridoo instrument. All subjects in this study received lessons at the Okayama branch.

## 2. Materials and Methods

### 2.1. Subjects

Subjects comprised 20 Japanese beginners (one male and 19 females, average 42.7 ± 12.7 years of age, median, 43 years of age) who started playing didgeridoo for the first time at the DHMPS Okayama branch. While all subjects lacked moderate to severe diseases, those with common diseases such as hypertension and diabetes mellitus were included. This study was approved by the Kawasaki Medical School Ethics Committee (Issue No. 2416, date of approval 13 June 2016). Among subjects recruited by the DHMPS Okayama branch and interested in playing didgeridoo, subjects of this study were approached verbally and in writing, and those from whom written consent was obtained were recruited as subjects in this study.

### 2.2. Actual Methods in DHPM

**Figure 2:**
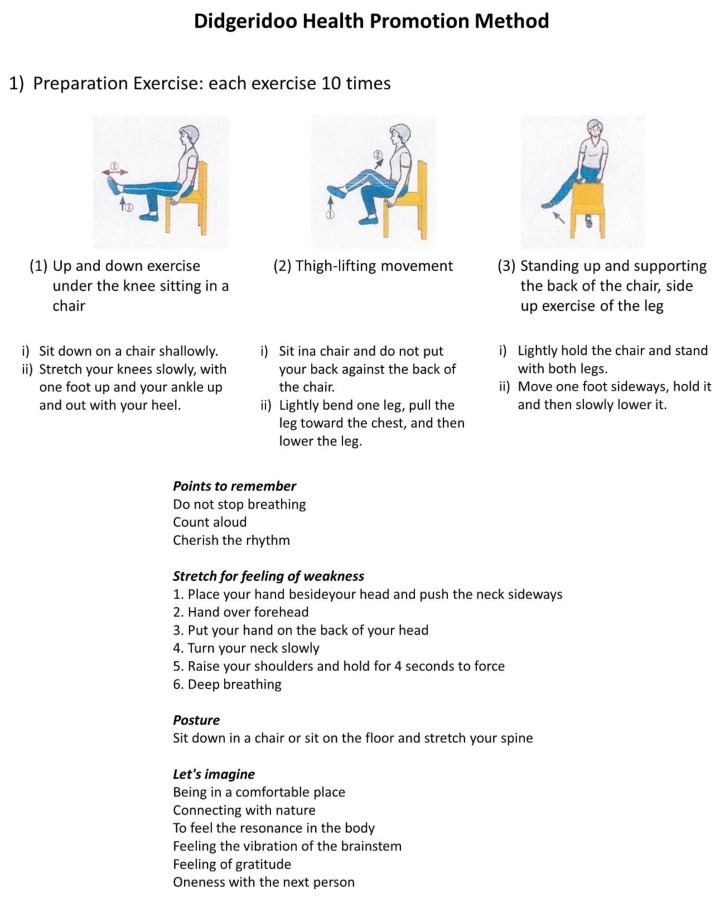
Preparation activity of the DHPM. An overview of the preparation activities of the DHPM. This is a 10-min preparatory movement, and while feeling the sense of oneness with nature and vibration of the brainstem, furthermore, feel the sense of oneness with the next person, loosen the whole body, relax the mind, and try to immerse yourself in the actual performance.

Figure 2 shows an overview of the preparatory movement of the DHPM. This was a 10-min preparatory movement. During these 10 min, subjects were encouraged to feel a sense of oneness with nature and the vibration of the brainstem, to feel a sense of oneness with the next person, to loosen the whole body, and to engage in mind relaxation. Thereafter, subjects tried to engage in an actual performance.

**Figure 3:**
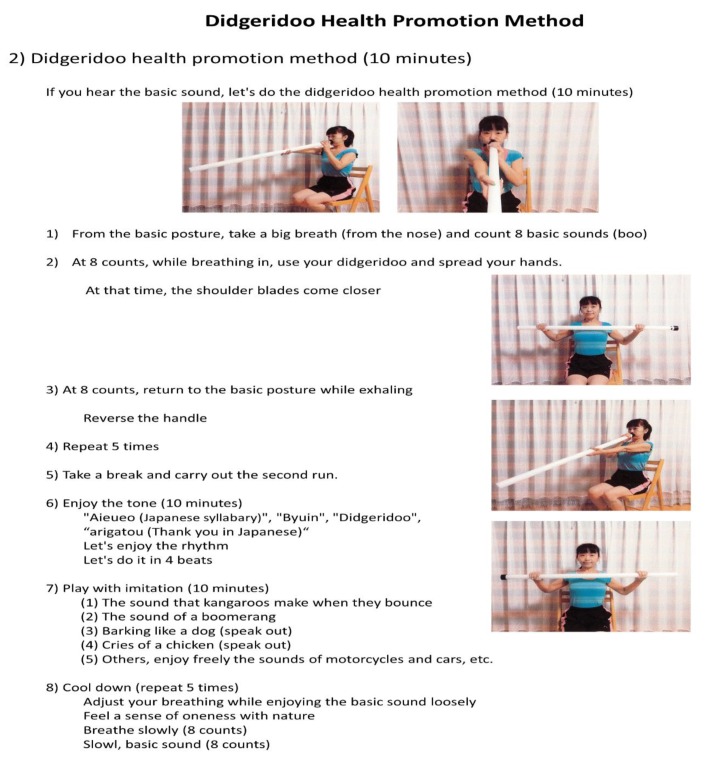
DHPM. After the preparatory movement, use of a plastic tube to imitate the didgeridoo to actually blow and make a sound and practice the health promotion method. An execution of a lesson.

Figure 3 shows details of the DHPM. Students generated sounds while playing with a plastic tube that imitated a didgeridoo. The details were as follows. Since this DHPM is not a performance involving the playing of a didgeridoo itself, the plastic tube is used as a tool in this health method, so the left and right arms (near the mouth, with arms extending forward to support the tube) are reversed. Additionally, students attempted to generate various sounds by movement of the lips. Finally, students cooled down and finished one lesson. It was a lesson that required about one hour to complete.

### 2.3. Research Schedule

**Figure 4:**
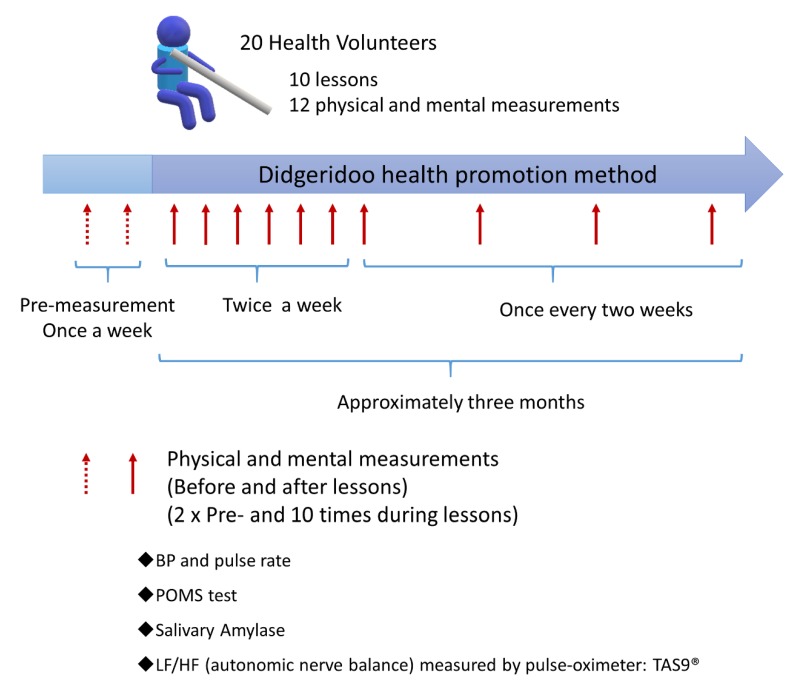
Lesson schedule and measurement of biological responses of subjects. Subjects who attended this study undertook 10 lessons of the DHPM, with the initial (intense) period of the course comprising two lessons a week (3 weeks, 6 lessons) and the final (more relaxed) period comprising lessons once every two weeks (8 weeks, 4 lessons). As a control, measurements of biological responses were performed twice prior to commencement of the entire course. The items measured as biological responses included mood surveys (using the Japanese version, Profile of Mood States 2nd Edition (POMS 2) (Kaneko Shobo Co., Ltd., Tokyo, Japan), blood pressure, and salivary amylase monitoring (Nipro Corporation, Osaka, Japan). The sAmy levels were measured using the salivary amylase monitor^®^ (Nipro dry clinical chemistry analyzer saliva amylase monitor^®^, Product Code 59-014). The autonomic nervous balance was measured using a TAS9VIEW^®^ pulse oximeter (YKC, Tokyo, Japan).

Figure 4 shows the lesson of the subject and the schedule employed for examination of the biological responses. Subjects that participated in this study undertook 10 lessons of the DHPM, with the initial (intense) period of the DHPM in the 10-week course comprising two lessons a week (3 weeks, 6 lessons), and the final (more relaxed) period comprising lessons once every two weeks (8 weeks, 4 lessons). As a control, measurements of biological response were performed twice prior to the commencement of the lessons (twice in one or two weeks). The items measured as biological responses included mood surveys (using the Japanese version of Profile of Mood States 2nd Edition (POMS 2) (Kaneko Shobo Co., Ltd., Tokyo, Japan), blood pressure using a home-use sphygmomanometer (OMRON upper arm type sphygmomanometer, HEM-8713, Kyoto, Japan), salivary amylase monitor^®^ (Nipro dry clinical chemistry analyzer saliva amylase monitor ^®^, Product Code 59-014, Nipro Corporation, Osaka, Japan), stress index by salivary amylase (sAmy) measurements, and autonomic nervous balance was measured, in addition to use of a TAS9VIEW^®^ pulse oximeter (YKC, Tokyo, Japan).

### 2.4. Survey of Mood (POMS2)

Investigation of mood was examined using POMS2 [12,13]. Although POMS2 is a descriptive questionnaire that conventionally determines the state of mood for the previous two weeks or so, in our study, the subject was asked to assess their mood state for that present moment.

The questionnaire comprised 30 questions, with answers that ranged in five stages from “not at all” to “very man.”. The “feeling condition” is was measured according to the following six scales.

T-A: Tension-Anxiety (Tension-Anxiety), “Feel tight/tensioned” comprised five stages. The higher the score, the more nervous a subject feels.

D: Depression-Depression (Depression), comprised five stages such as “the feeling is down and dark.” Higher scores indicate increased loss of confidence.

A-H: 5 stages such as Anger-Hostility and “Bad Mood”. A higher score indicates increased anger.

F: Comprised 5 stages such as “Fatigue” and “I get tired”. A higher score indicates an increased feeling of being tiredness.

C: Confusion, five items such as “Confusion”. A higher score indicates increased confusion and less thought.

V: Vigor (Vigor), 5 items such as “Vivid”. As this item is a positive item, unlike the other five scales, a lower score indicates that the activity is lost.

Total Mood Disturbance (TMD) was also calculated. This is a comprehensive expression of negative mood state (calculation formula below). The higher the main score, the more negative the mood, determined as follows: TMD = {[anger-hostility] + [confusion-embarrassment] + [depression-depression] + [fatigue-lethargy] + [tension-anxiety]} − [vigor-vitality]

### 2.5. Blood Pressure and Pulse Rate

Blood pressure and pulse rate were measured at resting, sitting on a chair, placing the arm at the height of the heart, and measuring mainly on the right forearm.

### 2.6. Saliva Amylase

The subjects first rinsed their mouths before the lesson, performed POMS2 questionnaire responses, had their blood pressure measured, followed by sAmy measurements [14,15]. After placement of the dedicated stick (Nipro saliva amylase monitor chip Product Code 59-010) under the tongue for 30 s, the concentration of Amylase was measured using a salivary amylase monitor^®^ according to the manufacturer’s instructions. The results were recorded on a special form.

### 2.7. Measurement of Autonomic Balance

Pulse waves were measured using a TAS9VIEW^®^ pulse oximeter (YKC, Tokyo, Japan). The measurement time was 3 min. In this device, by analyzing changes in the volume of arterioles at the fingertips with a waveform, a large number of indices can be displayed based on the average waveform type and the fluctuation width of the pulse interval. From the time series data of fluctuation, the high frequency fluctuation component (HF component) corresponding to respiratory fluctuation and low frequency component (LF component) corresponding to the Mayer wave, which relates to blood pressure fluctuation, were extracted. The autonomic nervous balance indicated by Ln[LF/HF] was used as an evaluation item [16,17]. According to the instruction manual of this device, normally, a value of less than 2.0 is defined as a “basic value”, 2.0 or more and less than 5.0 as “attention”, and 5.0 or more as “stronger attention”. In these cases, since “attention” and “stronger attention” are the ratio of LF/HF, the balance between the sympathetic nerve expressed by LF and the parasympathetic nerve of HF is high, that is, we have sympathetic dominance and the degree of tension and stress is high.

### 2.8. Statistical Analysis

Statistical analyses were performed using SPSS version 22 (IBM, Chicago) or Microsoft Office Excel 2013. A significant difference was shown if the degree of risk was less than 5% (*p* < 0.05).

## 3. Results

### 3.1. Evaluation of Mood: POMS2

**Figure 5:**
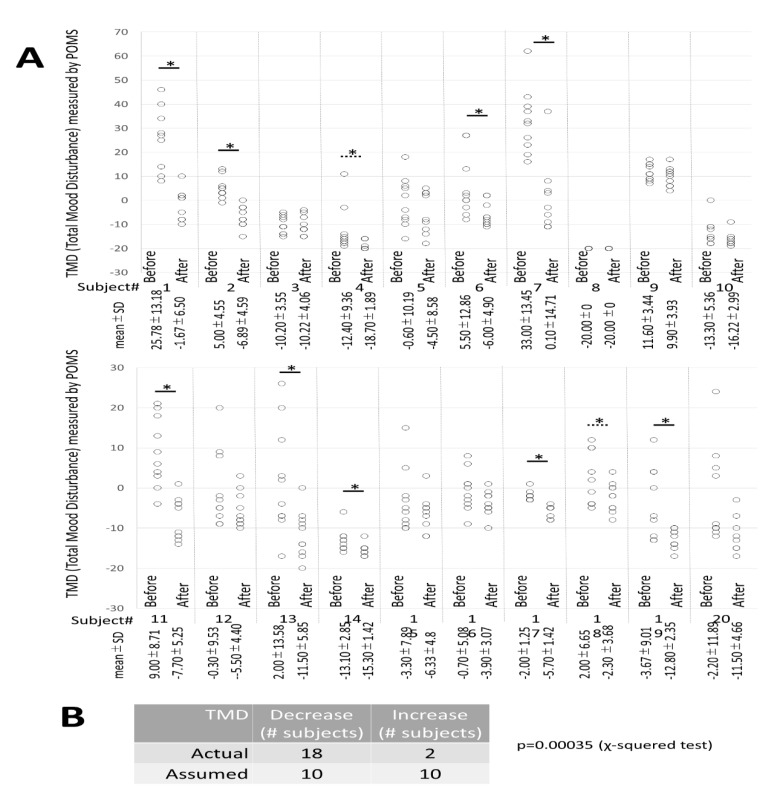
TMD from POMS2 Questionnaire. (**A**) The mean ± standard deviation of TMD before and after 10 lessons of the 20 individual subjects is shown. Eight out of twenty subjects showed significantly lower values after the lesson compared with values before the lesson. Two subjects showed a decreasing tendency (0.1 > *p* > 0.05). (**B**) A comparison was not made between groups before and after the lesson, but the number of people whose values after and before or after before and after were compared. Assuming that the predicted value would decrease or rise by half, the results indicated a decrease in 18 subjects and a rise in 2 subjects. In the chi-squared test, there were many subjects who dropped significantly (*p* = 0.00035).

Figure 5A shows the mean and standard deviation of TMD before and after 10 lessons for each of the 20 subjects. There were eight subjects with significantly lowered TMD, and two subjects with p values between 0.05–0.1 were judged as “tended to decline”. Additionally, 18 subjects fell, while 2 subjects rose in terms of whether the averaged TMD value decreased or increased after the lesson compared to that before the lesson, and not the statistical comparison between ‘before’ and ‘after’ lessons. As shown in Figure 5B, if it is assumed that values would decrease or increase by half, 18 subjects showed a decrease while 2 subjects showed an increase in measured values, which are significantly lower in the chi-squared test compared to this assumption.

A decrease in TMD can be understood as a decrease in negative mood, and these results indicate that taking the DHPM lessons results in improved mood state.

**Figure 6:**
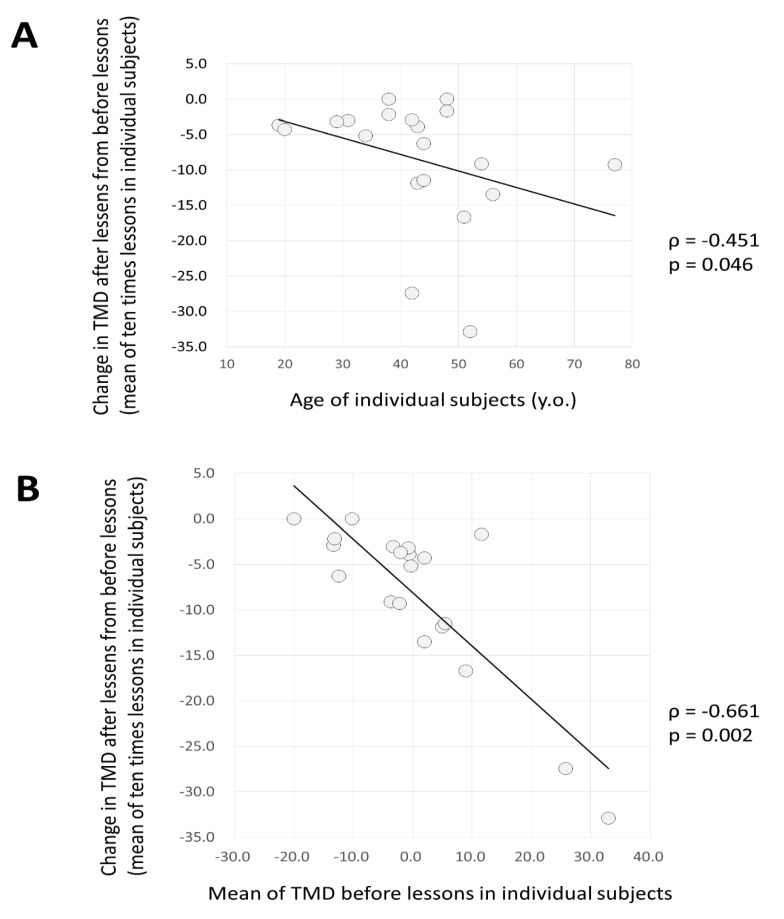
The relationship between the change in TMD (before and after the lesson) and the age, and the TMD before the lesson. (**A**) Relationship between the change in TMD before and after 10 lessons in the 20 subjects (post-value minus pre-value) and age. Results showed a significant negative correlation. (**B**) Relationship between change in TMD before and after 10 lessons in the 20 subjects (post-value minus pre-value) and TMD values of pre-lessons. This showed a significant negative correlation.

The relationship between the change before and after a lesson (post-value minus pre-value) of the TMD (measured before and after 10 lessons in 20 subjects) is shown in Figure 6. The relationship between the age (Figure 6A) and the average value of the TMD before the lesson is also shown (Figure 6B). All showed a significant inverse correlation.

The results suggested that the older the subject, the greater the decrease in TMD after a lesson, and the greater the TMD before a lesson, the greater the degree to which the TMD declines after a lesson.

As for age, considering that older people generally have higher TMD (the negative factor in mood is stronger) than younger people, the DHPM is assumed to be useful for elderly people from the viewpoint of mood.

### 3.2. Blood Pressure and Pulse

Blood pressure was compared before and after lessons or twice before the entire lessons began and after the entire lessons ended. However, no particular change was observed.

**Figure 7:**
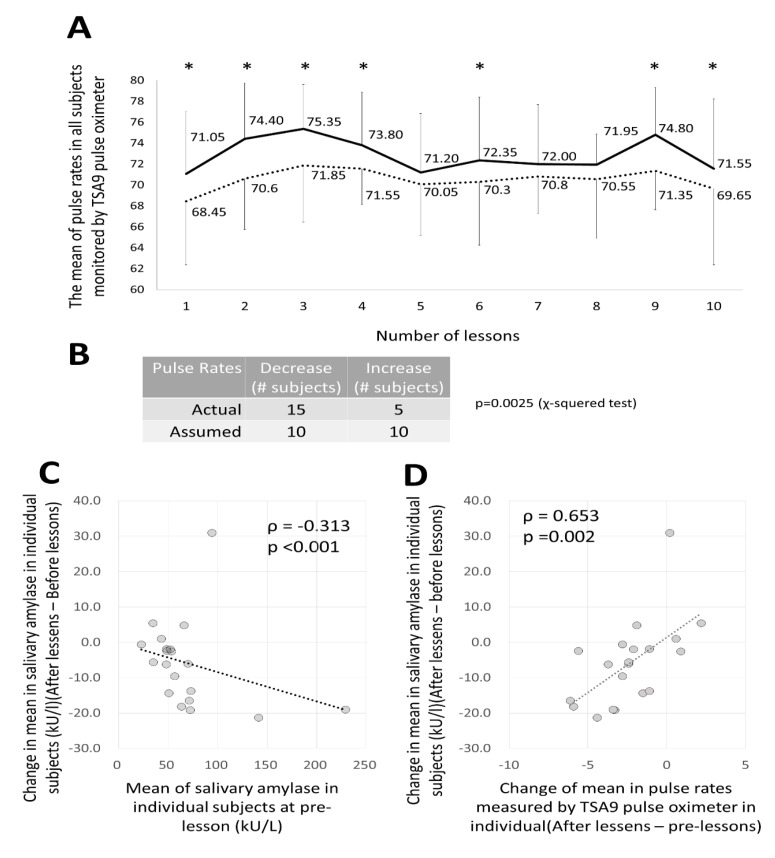
Pulse changes in subjects before and after lessons and changes in salivary amylase levels that serve as stress indicators. (**A**) Changes in pulse rate as measured by TAS9VIEW^®^. The horizontal axis shows the first to the tenth lesson. The solid line shows the average pulse rate of the 20 subjects before each lesson. The dotted line represents the average value after the lesson. The pulse rate decreased significantly, except at the fifth, seventh, and eighth lessons. (**B**) The average pulse rate for all of the 10 lessons of the 20 subjects: the pulse rate declined in 15 subjects and rose in 5 subjects. The chi-squared test was performed with each half as a predicted value. There were many subjects whose value decreased significantly. (**C**) For salivary amylase, the horizontal axis shows the average value before each lesson of the 20 subjects, and the vertical axis shows the change in the average values (post-value minus pre-value) before and after the lessons of these subjects. This shows a significant inverse correlation. (**D**) Horizontal axis: change in pulse rate value of the 20 subjects as measured by TAS9VIEW^®^ (post-value minus pre-value); vertical axis: change in salivary amylase value before and after lessons of the subjects (post-value minus pre-value). This shows a significant positive correlation.

In Figure 7A, the fluctuation in the average value of the pulse-rate of the 20 subjects as measured by TAS9VIEW^®^ is shown before and after each of the lessons. Interestingly, in all but three (5th, 7th, and 8th) lessons, the pulse was significantly lower after the lesson than before the lesson. Furthermore, when comparing the average values before and after the lessons for all 20 subjects, 15 subjects experienced a decline while 5 subjects experienced a rise. Assuming that the decrease and rise comprised 10 each, it was determined that the drop was significantly higher in the chi-squared test (Figure 7B).

The results from these pulse values suggested that the pulse would be reduced with the DHPM lessons, which is predicted to become more parasympathetic in nature. It is assumed that it has a calming effect along with the tendency of TMD to decline in the survey of mood.

### 3.3. Stress Index: sAmy

With regard to sAmy levels, we examined changes before and after a lesson, in subjects as a whole, or individually, with no apparent difference being observed.

However, as shown in Figure 7C, in individual cases, subjects who showed a higher mean of pre-lesson values (*X*-axis) showed a greater decrease in the average changes (post-value minus pre-value) of sAmy in 10 lessons (*Y*-axis). This showed an inverse correlation with significant difference. This result indicates that the higher the stress level before the lesson, the more readily the stress relief effect of the lesson appears.

Furthermore, as shown in Figure 7D, the correlation between the mean value of the changes (post-value minus pre-value) in sAmy for the entire 10 lessons of each subject and the pulse changes (post-value minus pre-value) indicates a positive correlation with statistical significance. The results show that subjects who showed greater decreases in stress index as determined by sAmy levels revealed greater decreases in pulse rate changes, which indicates the predominance of the parasympathetic effect. Thus, it was found that the stress reduction effect of the DHPM is inferred from the results of sAmy and pulse rate.

### 3.4. Autonomic Nerve Balance: Ln[LF/HF]

**Figure 8:**
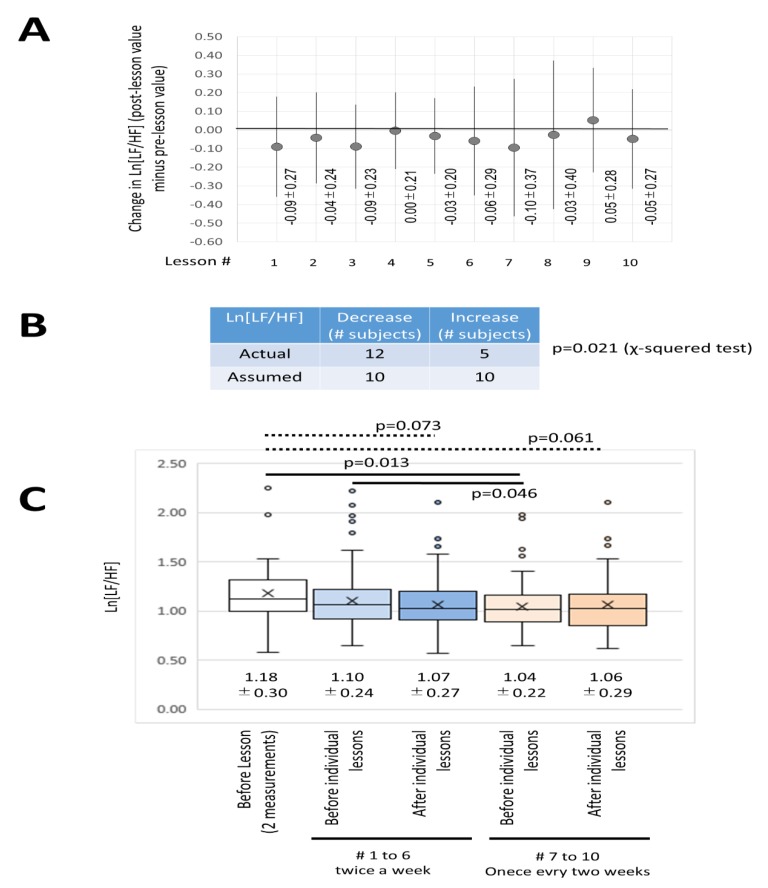
The effect of the DHPM on autonomic balance. (**A**) Average value of changes in Ln[LF/HF] of the 20 subjects in 10 lessons (post-value minus pre-value). The values declined except in the case of lessons 4 and 9. (**B**) Chi-squared test assuming that the results of panel A are predicted to have a decline in half and a rise in half. There were many subjects that showed a significant decrease (*p* = 0.018). (**C**) In the 10 lessons of the 20 subjects, 12 subjects showed decreased Ln[LF/HF] more often after the lesson than before the lesson, 3 subjects remained unchanged, and 5 subjects showed increased Ln[LF/HF]. A chi-squared test was performed with half of the expected change decreasing and half increasing. Many subjects showed significant decreases (*p* = 0.021). (**D**) In an effort to observe the long-term effects of the DHPM, the average, median, and distribution of Ln[LF/HF] of the 20 subjects are shown using a box and whisker plot. From the left, 2 measured values before entering the lesson (row 1), followed by the 1st to 6th measurements, that is, before (row 2) and after (row 3) the lesson when the lesson (the initial (intense) period) is performed twice a week (6 lessons total) and the value before the lesson (row 4) and after value (row 5) of the 7th to 10th lessons (one lesson every 2 weeks, 4 lessons total: the final (more relaxed) period). The solid line indicates the significant difference (*p* < 0.05), and the dotted line indicates the trend (0.1> *p* > 0.05).

The autonomic nervous balance was measured with Ln[LF/HF] as calculated by TAS9VIEW^®^. As shown in Figure 8A, the average value of Ln[LF/HF] variation (post-value minus pre-value) of 20 subjects in 10 lessons decreased, except for the 4th and 9th lessons. If it is assumed that half of the lessons will result in a drop and the other half in a rise, then the results will be significantly different in the chi-squared test as shown in Figure 8B. This result indicated that the predominant effect of the DHPM was related to the parasympathetic nerve. Furthermore, as shown in Figure 8c, the number of times the decrease or increase in Ln[L/HF] was greater for the 10 lessons was examined. The results indicated that 12 subjects showed decreased Ln[LF/HF] more often after the lesson than before the lesson, 3 subjects remained unchanged, and 5 subjects showed increased Ln[LF/HF]. If half of the subjects were assumed to fall and half were to rise, the chi-squared test significantly showed that the number of subjects who showed decreases was large.

Additionally, the aforementioned analyses were performed for the entire group of 10 lessons or all 20 subjects, with the initial (intense) period of the DHPM in the 10-week course comprising two lessons a week (3 weeks, 6 lessons) and the final (more relaxed) period comprising lessons once every two weeks (8 weeks, 4 lessons). This implementation of intense and more relaxed lesson periods was designed to determine whether the effects on the autonomic nervous system caused by the DHPM were sustainable, as determined by Ln[LF/HF].

As shown in Figure 8D, the pre-values of Ln[LF/HF] for each lesson in the 20 subjects during the final (more relaxed) lessons (7th to 10th lessons) were significantly lower compared to the two control measurements determined prior to the commencement of the first lesson. Furthermore, the pre-values of Ln[LF/HF] for each lesson in the 20 subjects during the final (more relaxed) period (7th to 10th lessons) were significantly lower compared with the pre-values of Ln[LF/HF] for each lesson in the 20 subjects during the initial (intense) period of the lessons (the 1st to 6th lessons). Additionally, post-values of Ln[LF/HF] for each lesson in the 20 subjects during the initial period (the 1st to 6th lessons) or the final period (7th to 10th lessons) tended to be lower compared with that observed prior to the commencement of the lessons.

These results suggested that continuous practice of the DHPM leads to stabilization of autonomic nervous balance and formation of a parasympathetic-dominant condition in daily life.

## 4. Discussion

Awareness of health promotion by the general public is increasing, and various health promotion methods are being advocated. At the first, the results and connection to similar studies will be discussed, then the reflection of the study will be summarized and finally applications of the research results into the area of health promotion suggested.

One of the authors, T. Kojima, became familiar with playing the traditional wooden brass instrument referred to as a didgeridoo and devised the DHPM (playing practice as a “health promotion method” including preparatory exercises). It was thought that by employing this method, in addition to strengthening respiratory muscles and other appropriate muscles and contributing towards positive mental aspects, stabilization of patients could be achieved. Therefore, the effects of the DHPM were observed and documented from the viewpoint of mood fluctuation, stress index (both mentally and physically), and autonomic nervous control.

Physical effects such as enhancement of respiratory muscles are also considerable after taking DHPM lessons. However, in this study series, lessons were performed in a small private room, and not in a hospital or laboratory. Although the spirometer is an effective instrument in measuring respiratory muscle strength, it was impossible to use this device in the private room where lessons were conducted. This is why we focused on mood, stress, and the autonomic nervous system in this study. Future investigations of respiratory muscle strength will be performed based on this study.

Mood change measured by POMS2 assumed that in 10 lessons, the negative mood would be mitigated and it would be possible to form a foundation to live in a vivid mood. This effect was greater in subjects who were older or had higher TMD prior to the lessons. The utility of the DHPM was observed for subjects who had a negative mood.

Furthermore, it was recognized that pulse balance was significantly reduced after the lessons and that the parasympathetic nerves were dominant even in the autonomic nervous balance observed in the pulse measurement. Additionally, as a result of monitoring the stress levels by the measurement of sAmy, although no clear result was obtained for each lesson or subject, it was found that the higher the value before the lesson, the greater the reduction before and after the lesson. Furthermore, this showed a positive correlation with pulse changes. That is, the value of sAmy, which is a stress indicator, also decreased together with the decrease in pulse rate after receiving lessons (the degree to which the parasympathetic nerves dominate). The fact that these several indicators show the same inclination supports the notion that the DHPM provides a stress-relieving action.

Additionally, regarding changes in the autonomic nervous system and considering the pulse as a heart rate monitor, the autonomic nervous balance was examined using a pulse oximeter and Ln[LF/HF] determined. The results also showed parasympathetic dominance in many of the lessons and in many subjects as well as other indicators. Furthermore, the DHPM implemented by subjects on this occasion consisted of a 10-week schedule, with the initial (intense) period comprising two lessons a week (3 weeks, 6 lessons) and the final (more relaxed) period comprising lessons once every two weeks (8 weeks, 4 lessons). In the final period, it became clear that the autonomic nervous balance as measured by Ln[LF/HF] became parasympathetic-dominant. These results suggested that each lesson and their continuation would lead to a state of parasympathetic dominance, that is, relaxation of stress, relaxation of excessive tension as a way of living, and stability.

DHPM has a mental or autonomic nervous control effect. Additionally, although it has not been verified in this study, physical effects such as the enhancement of respiratory muscles are also assumed. From these results, it is thought that the DHPM should be recommended as one means of contributing towards health promotion in both mind and body.

There are some limitations of this study. One of these concerns the absence of a control group due to the difficulties involved in enrolling healthy people with similar backgrounds to non-control subjects in terms of life-style, eating habits, physical training status, and other parameters. This is why measurements were performed twice before the commencement of DHPM training. Furthermore, when obtaining informed consent, we explained the aim and purpose of this study, the nature of the instruments to be employed, and what these instruments might reveal in this study. Thus, subjects were essentially aware that DHPM might influence mood, stress, and the autonomic nervous system. In an effort to minimize or avoid the effects of this awareness on experimental outcomes, parameters were measured twice before subjects commenced with DHPM lessons.

In this study, it was verified that DHPM induces mood improvement, stress reduction, and parasympathetic dominance of the autonomic nervous system, even with the above limitations. This is a health promotion method that can be fully evaluated in terms of the promotion of mental health and emotional well-being. How can this method be applied to daily life? The DHPM comprises one lesson twice a week for the first two weeks, and after that time lesson frequency can drop to once every two weeks. As shown in Figure 1C, classroom sizes preferably comprise small groups of up to about 20 people. Lessons will usually be completed within about one hour or in less time for experienced participants. Given this situation, it is possible to set up a classroom at lunchtime at a workplace or after working hours. In recent occupational health considerations, the importance of primary prevention and further prevention of mental health disorders has been emphasized [4,5,6]. Furthermore, rehabilitation of individuals with mental health problems and return to work as tertiary prevention strategies are also issues. It would be beneficial to introduce this method for the purposes of primary prevention and tertiary prevention. Furthermore, by establishing DHPM classes within the local community, it will help to improve communication among residents, and the level of individual health throughout the region will increase. These aims are consistent with the proposed concept.

The didgeridoo health method promotion society is also focusing on the training of leaders (Meisters) who will teach the DHPM. The implementation of this health method is also appropriate for lesson groups comprising a few to about twenty people (Figure 1C), and the training of Meisters is also necessary for its continued implementation. Henceforth, in addition to targeting the general healthy public, the DHPM can be implemented by companies and factories as a form of health promotion. Workers in various industries and factory settings have recently been encouraged to engage in physical activities at the gym. Gazmararian et al. evaluated the effectiveness of addressing multiple barriers to physical activity (PA) using interventions at the workplace. Their results showed that there was no significant improvement when a group was involved in gym activity alone compared to the control. However, with education based on physical training in the gym, improvements were sustained for over 9 months [18]. Partonen et al. reported that supervised physical exercise combined with exposure to bright light appears to be an effective intervention for improving mood and certain aspects of health-related quality of life in the winter time [19]. Since their report dealt with subjects in Finland, bright lights might be an important factor in domestic circumstances. However, a combination of physical exercise and mental health promotion is certainly better for total health promotion. Additionally, if these workers performed DHPM twice a week, it would enhance their physical as well as their mental health. Another appealing aspect of DHPM concerns the involvement of regional neighborhood associations/communities, where DHPM lessons allow for up to approximately twenty people to participate concurrently. Thus, it is better to begin with small groups in regional communities and then spread to other community groups. These efforts should assist in facilitating health promotion using the didgeridoo.

In the future, the health promoting effects of DHPM should be examined in comparison with other resembling methods. We believe the DHPM is unique because of improving mental as well as physical status, although physical changes were not examined in this study with limitation of location of DHPM. There are many active health promotion methods such as jogging, training at the gym for physical status. In addition, some nutrition, oral supplements such as vitamins, and scant meals are also provided. To compare these methods and DHPM, DHPM may possess advantage for costs; however, it needs Meister/teachers for training and location since DHPM makes not small sounds. Once people experienced DHPM, they may take care of their physical and mental health more than before. This may make them be much healthier.

## 5. Conclusions

The DHPM was developed and evaluated regarding mood, mental stress, and stability of autonomic nervous system. As a result, the subjects who completed the 10-week DHPM revealed improvement of mood. In addition, stresses measured by sAmy decreased after the lessons with increasing age of the subject. Moreover, it has been shown that autonomic nerve system tended toward parasympathetic dominant over the 10 weeks, and it was also clear that the effect was sustained. Taken together, the DHPM is an adequate tool for health promotion. Expansion and distribution of the DHPM for work places as well as regional neighborhoods should be considered.
